# Production of minor ginsenosides by combining *Stereum hirsutum* and cellulase

**DOI:** 10.1371/journal.pone.0255899

**Published:** 2021-08-06

**Authors:** Wenhua Yang, Jianli Zhou, Jean Damascene Harindintwali, Xiaobin Yu

**Affiliations:** 1 Key Laboratory of Carbohydrate Chemistry & Biotechnology, Ministry of Education, School of Biotechnology, Jiangnan University, Wuxi, Jiangsu, China; 2 Key Laboratory of Industrial Biotechnology, Ministry of Education, School of Biotechnology, Jiangnan University, Wuxi, Jiangsu, China; 3 School of Food and Drug Manufacturing Engineering, Guizhou Institute of Technology, Guiyang, Guizhou, China; ICAR-Directorate of Mushroom Research, INDIA

## Abstract

Minor ginsenosides (MGs) (include ginsenoside F_2_, Compound K, PPT, etc), which are generally not produced by ginseng plants naturally, are obtained by deglycosylation of major ginsenosides. However, the conventional processes used to produce deglycosylated ginsenosides focus on the use of intestinal microorganisms for transformation. In this study, an edible and medicinal mushroom *Stereum hirsutum* JE0512 was screened from 161 β-glucosidase-producing soil microorganisms sourced from wild ginseng using the plate coloration method. Furthermore, JE0512 was used for the production of CK from ginseng extracts (GE) in solid-state fermentation (SSF) using 20 g corn bran as substrate, 4 g GE, and 20% inoculation volume, and the results showed that the highest CK content was 29.13 mg/g. After combining *S*. *hirsutum* JE0512 with cellulase (*Aspergillus niger*), the MGs (F_2_, CK, and PPT) content increased from 1.66 to 130.79 mg/g in the final products. Our results indicate that the *Stereum* genus has the potential to biotransform GE into CK and the combination of *S*. *hirsutum* JE0512 and cellulase could pave the way for the production of MGs from GE.

## 1. Introduction

Ginseng is a perennial herbaceous plant of the genus *Panax* in the Araliaceae family. Among them, Asian (*Panax ginseng*) and American ginseng (*Panax quinquefolius*) are the two most well-known species and are both commonly used for their medicinal properties [1]. Ginseng is a famous and valuable medicinal and edible plant; this perennial herb has been used to enhance immunity, reduce fatigue, slow aging, and provide nutrition around the world [2]. Modern pharmacological experiments have shown that ginseng contains a variety of bioactive compounds, including ginsenosides, polysaccharides, phenols, and polyacetylenes [3]. Among them, ginsenosides are considered to be the main components underlying the pharmacology and biological activity of ginseng [2].

**Fig 1:**
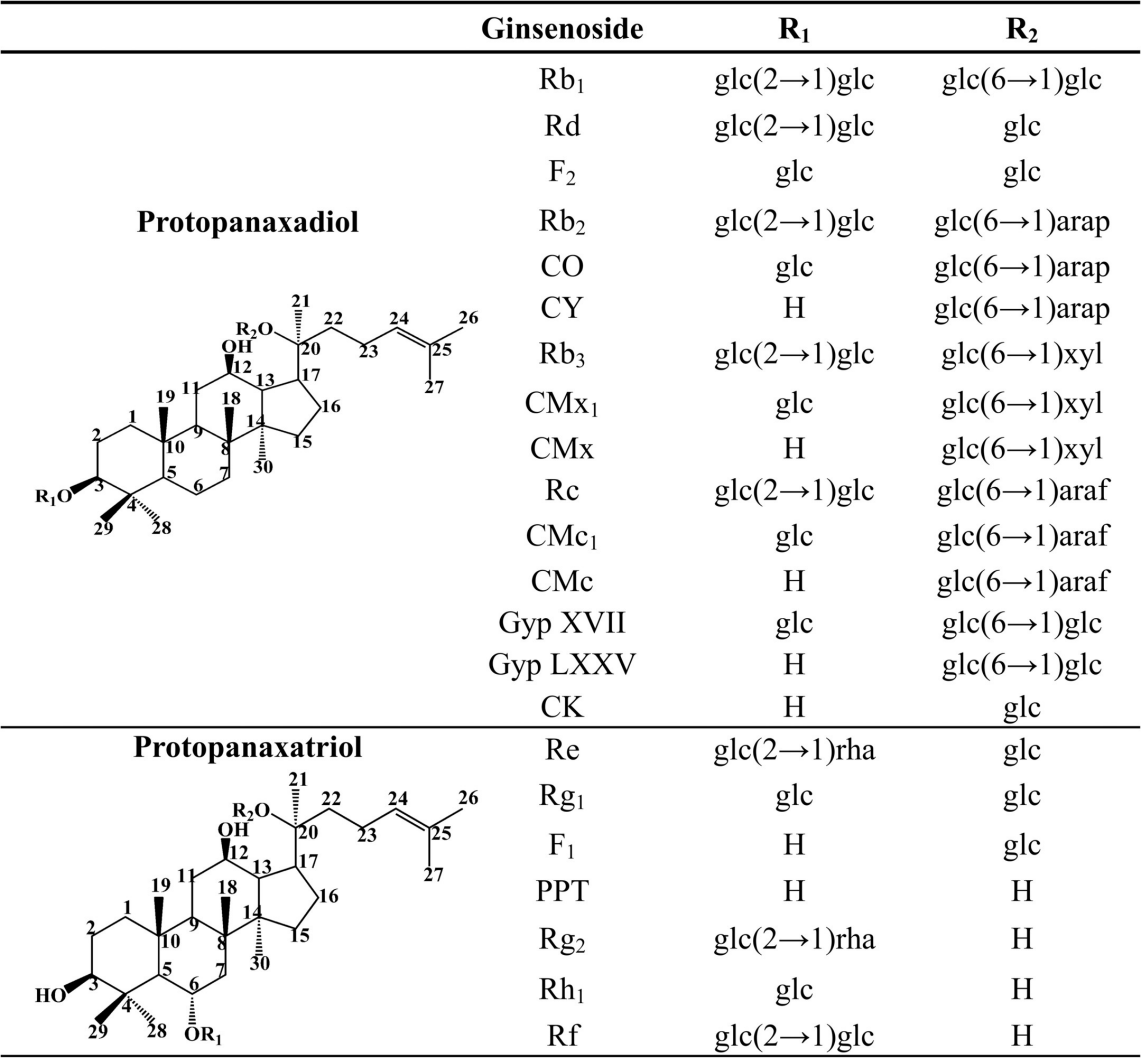
The chemical structural formula of ginsenosides. Glc, β-D-glucopyranosyl; arap, α-L-arabinopyranosyl; xyl, β-D-xylopyranosyl; araf, α-L-arabinofuranosyl; rha, α-L-rhamncpyranosyl; Gyp, gypenoside; C, compound.

As of the time of writing, about 180 ginsenoside derivatives have been identified from different parts of *Panax quinquefolius*, processed *P*. *ginseng* products, and other *Panax* spp. [4]. All ginsenosides share the same basic structure, consisting of a dammarane steroid nucleus with 17 carbon atoms arranged in four rings. The structural diversity of ginsenosides is due to differences in the type, attachment position, and the number of sugar moieties (Fig 1). Ginsenosides are classified into three groups based on the types of aglycone structures; namely, oleanane (Ro), protopanaxadiol-type (Rb_1_, Rb_2_, Rb_3_, Rc, Rd, F_2_, Compound K, and PPD), and protopanaxatriol-type (Re, Rf, Rg_1_, Rg_2_, Rh_1,_ and PPT) [5]. MGs [6,7] are more pharmacologically active due to their lower molecular weight, higher cell membrane permeability, and easier absorption in the gastrointestinal tract compared to the major ginsenosides [8]. Recent *in vivo* and *in vitro* studies have shown that MGs have many pharmacological activities, including anti-tumour [9], anti-inflammation [10,11], anti-cancer [12,13], skincare [14], and hypoglycaemic [15] properties. Among them, Ginsenoside Compound K (CK) tablets are undergoing clinical trials in China for arthritis prevention and treatment under approval (CDEL20130379) by the China Food and Drug Administration (CFDA) [16]. We used CK content as the main indicator for screening microorganisms because of the high pharmacological value of CK.

MGs, which mostly do not exist naturally in ginseng plants, are obtained by deglycosylation of major ginsenosides (such as Rb_1_, Rb_2_, Rc, Rd, Re, Rg_1_) [17]. A plethora of techniques, including heat treatment [18], acid or alkaline hydrolysis [18], enzyme transformation [2], and microbial transformation [19] have been developed to produce MGs from major ginsenosides. Microbial and/or enzymatic treatment provides many benefits over conventional physical and chemical treatments, as they involve environmentally-friendly processes that can be performed under mild conditions with high regio- and diastereoselectivity and in simple reaction steps [20]. Usually, in these methods, a large number of microbial or enzyme preparations are obtained by liquid-state fermentation (LSF). However, in the past few years, compared with LSF, SSF has become a promising alternative for obtaining higher enzyme titers and biological products from low-value raw materials [21]. SSF, using different agro-industrial wastes as both carbon sources and inducers for microbial transformation, would make the MGs production process more economically viable. In recent years, few studies have reported that some edible and medicinal mushrooms could possess a series of ginsenoside-hydrolyzing β-glucosidases and can effectively transform ginsenoside Rb_1_ to CK [20,22]. In addition, many researchers have focused on using purified monomer ginsenosides instead of GE for the production of CK, which would increase production costs at an industrial scale.

Driven by the root exudates, rhizosphere soil accommodates distinct microbial species with diversified activities. Many β-glucosidase producing microorganisms have been isolated from ginseng field soils and proved effective in hydrolyzing ginsenosides [23,24]. In this study, an edible and medicinal mushroom *Stereum hirsutum* JE0512 was isolated to transform GE (containing a variety of major ginsenosides) into MGs. To the best of our knowledge, there have been no prior studies on the use of *S*. *hirsutum*, a producer of β-glucosidase, to produce MGs from GE through SSF. As cellulase contains β-glucosidase, which is involved in the production of MGs [25], we hypothesized that the combination of cellulase and *S*. *hirsutum* JE0512 could enhance MGs production. In this study, we focused on optimizing the SSF process parameters (agro-industrial substrate, inoculation volume, amount of GE, and fermentation period) for CK production and the changes in ginsenoside content during *S*. *hirsutum* JE0512 SSF with two different cellulases. The production of ginsenosides through SSF not only reduces the production cost associated with the conventional process but also provides a reference for the production of high value-added products using microbial/enzyme stage control.

## 2. Materials and methods

### 2.1. Materials

Ginsenosides standards (Rb_1_, Rb_2_, Rb_3_, Rc, Rd, F_2_, CK, Re, Rg_1_, F_1_, and PPT) were purchased from Chengdu Must Biotechnology Co., Ltd. (Chengdu, Sichuan, China), with a purity of 98%. GE (containing 80% ginsenosides) from the stems, leaves, and roots of *Panax ginseng* was collected from Xi’an Tianxingjian Natural Bio-products Co., Ltd. (Xian, Shanxi, China). Other chemicals and reagents used were of analytical grade and were purchased from Sigma-Aldrich (Shanghai, China).

### 2.2. Screening of β-glucosidase producing strains

Genipin, the product of geniposide hydrolysis by β-glucosidase, can react with amino acids to form a stable blue-colored compound [26]. Based on this principle, we designed a screening medium for isolating β-glucosidase-producing fungal strains. Ginseng rhizosphere soil samples were collected from 5 cm^3^, 20 cm^3^, and 1 m^3^ around wild ginseng plants in Fusong (Jilin, China), stored at 4°C and used within half a year. 1g soil sample was added to 9ml of deionized water and diluted step by step. Diluted samples (200 μL) with dilution factors of 10^−1^ to 10^−6^ were spread on modified potato dextrose agar (PDA) medium, supplemented with 1 g/L geniposide and 10 g/L L-glutamate, and cultured at 25°C for 3–7 days. Based on visual inspection of the colony growth and color change on the plate, all colonies that changed the color of the medium were picked and cultured on new plates for purification. The pure isolates were stored at 4°C for further experiments.

### 2.3. Identification of CK producing strains

The selected fungal strains were transferred to a 250-ml Erlenmeyer flask containing 50 ml PDA medium and cultured at 25°C in a rotating shaker (180 rpm). After 4 days, 1 g of GE was added to the growth medium, and the cultures were further incubated for 7 days. The fermentation broths were harvested by centrifugation (5000 ×*g*, 10 min) for CK content assays, which were determined by HPLC. The fungal strain with the highest CK content was selected for further characterization. For molecular characterization, total genomic DNA was extracted using the Ezup column fungal DNA kit (Sangon Biotech, Shanghai, China) according to the manufacturer’s instructions. The fungal 18S ribosomal DNA (rDNA) universal primers 5´- GTAGTCATATGCTTGTCTC-3´ and 5´-GCATCACAGACCTGTTATTGCCTC-3´ were used as templates, and the PCR products were recovered using the SanPrep column DNAJ gel recovery kit (Sangon Biotech, Shanghai, China) and sent to Sangon Biotech (Shanghai, China) for sequencing. The 18S rDNA sequence was compared against the GenBank database using the online BLAST program (http://www.ncbi.nlm.nih.gov/BLAST/Blast.cgi). A phylogenetic tree was constructed using the neighbor-joining method with the application MEGA 7.0 [27].

### 2.4. Analysis and optimization of multiple factors for SSF

For the selection of an optimal agro-industrial substrate, different substrates such as rice, corn flour, soybean bran, corn bran, wheat malt, oat, soybean flour, and wheat were tested. Briefly, 20 g of the substrate was mixed with 1 g GE at a moisture content of 60% and sterilized in a 250-ml Erlenmeyer flask. Then, samples were inoculated with the selected fungal strains and grown at 25°C, pH (6.8) in the dark for 5 days through SSF.

Factors such as inoculation volume (v/w) (5%, 10%, 15%, 20%, 30%), amount of GE (1 g, 2 g, 4 g, 7 g, 10 g), and fermentation period (0 d, 3 d, 5 d, 8 d, 10 d, 15 d) were optimized by varying one factor at a time, and the optimized parameters were subsequently used to evaluate the synergistic effect of the combination of fungal strain and cellulase on the production of ginsenosides.

### 2.5. Combination of JE0512 with cellulase for SSF

The optimal fermentation method for the production of CK was selected based on the optimization results in 2.4. Cellulases derived from *Trichoderma viride* and *Aspergillus niger* were separately inoculated into the sterilized fermentation medium at a ratio of 2.5% (w/w) and fermented using the optimal fermentation method in 2.4 to compare the effect of adding different cellulases on the composition of ginsenosides. The enzyme activity of solid cellulase from *T*. *viride* (purchased from Sinopharm, Shanghai) was 15,000 U/g and the enzyme activity of solid cellulase from *A*. *niger* (laboratory prepared and preserved) was 110 U/g.

### 2.6. Sample preparation

For determination of β-glucosidase activity, the fermentation products were extracted with Na_2_HPO_4_-NaH_2_PO_4_ buffer (pH 6.0, 50 mM) in a 1:5 ratio at 4°C for 12 h, and the extracted solutions were centrifuged (5000 ×*g*, 20 min) to filter the mycelium and impurities. The supernatants were collected as crude extracts [28].

To determine ginsenoside content, the fermentation products were extracted with an equal volume of n-butanol, and the n-butanol fraction was then evaporated until dry using a vacuum rotary evaporator. A total of 10 ml of methanol (HPLC grade) was added to the dried products, and the solutions were filtered using the 0.22-μm ultrafiltration membrane. HPLC and UPLC-Q-TOF-MS analysis were performed on the resulting product.

### 2.7. Analysis of β-glucosidase activity

The β-glucosidase activity was assayed as described by the previous reports [3,29], with some modifications. 100 μL crude enzyme extracts were mixed with 200 μL of 5 mmol/L PNPG and reacted at 50°C. After 30 min of reaction, 2 ml of 1 mol/L Na_2_CO_3_ was added to terminate the reaction, and absorbance data were obtained using a microplate spectrophotometer at a wavelength of 400 nm. P-nitrophenol (p-NP) was used for the preparation of the standard curve, and one activity unit (AU) of β-glucosidase was defined as the amount of enzyme required to release 1 μmol of p-NP per min under assay conditions.

### 2.8. Quantitative and qualitative analysis of ginsenosides

Samples of ginsenoside standards (Rb_1_, Rb_2_, Rb_3_, Rc, Rd, F_2_, CK, Re, Rg_1_, F_1_, and PPT) and the fermentation samples were quantified using an Agilent 1260 HPLC system (Agilent Technology, California, USA), equipped with a Sepax GP-C18 column (4.6 mm × 250 mm, 5 μm). Ginsenoside standard solutions with concentrations of 0.5, 1, 1.5, 2, and 4 mg/mL were prepared in methanol (HPLC grade). Acetonitrile (solvent A) and water (solvent B) were used as the mobile phases. Gradient elution was performed, beginning with 70% solvent A and 30% solvent B for 0–30 min, progressing to 45% solvent A and 55% solvent B for 30–45 min, and 70% solvent A and 30% solvent B for 45–60 min. The flow rate of the mobile phase was 1 mL/min, the column temperature was 30°C, injection volume was 10 μL, and this was monitored at 203 nm absorbance using a UV spectrophotometric detector. In addition, samples of the ginsenoside CK standard and the fermentation samples of the strain with the highest yield of CK were analyzed using MALDISYNAPTQ-TOFMS (Waters Acquity UPLC, Mass, USA).

The different ginsenoside content standard curves were defined using the equations, Rb_1_: Y = 4040.9X - 114.63, R_2_ = 0.999; Rc: Y = 2706.4X - 1.9358, R_2_ = 1.000; Rb_2_: Y = 2695.5X + 63.511, R_2_ = 0.999; Rb_3_: Y = 2705.1X + 47.813, R_2_ = 0.999; Rd: Y = 2781.3X + 79.36, R_2_ = 0.999; F_2_: Y = 3665.6X + 99.908, R_2_ = 0.999; CK: Y = 4332.1.5X - 60.342, R_2_ = 0. 999; Re: Y = 2881.7X + 95.954, R_2_ = 0. 999; Rg_1_: Y = 3292.2X + 18.353, R_2_ = 0. 999; F_1_: Y = 4219.5X + 33.093, R_2_ = 0. 999; PPT: Y = 1480.6X + 30.957, R_2_ = 0. 999, where Y is the peak area and X is the ginsenoside content (mg/mL).

Ginsenoside content was subsequently calculated using according to the following Eq (1).

$${\text{Y}}_{\text{GC}}=\frac{X\times V}{m\times 80\%}$$ (1)

where Y_GC_ is the ginsenoside content in the sample in mg/g; X is the ginsenoside content in mg/ml; V is the volume of methanol (HPLC grade) added to the dried products in mL; m is the mass of GE in g.

### 2.9. Statistical analysis

All findings are reported as the mean ± standard deviation (n = 3). Data analyses were conducted using SPSS 25.0 [19] and results are expressed as mean ± standard deviation. One-way ANOVA was used to assess the statistical significance of the differences in expression levels. Different letters (a-e) indicate significant differences between each other (P < 0.05). Graphs and plots were generated using Origin 9.1.

## 3. Results and discussions

### 3.1. Isolation and screening of β-glucosidase producing fungi

**Fig 2:**
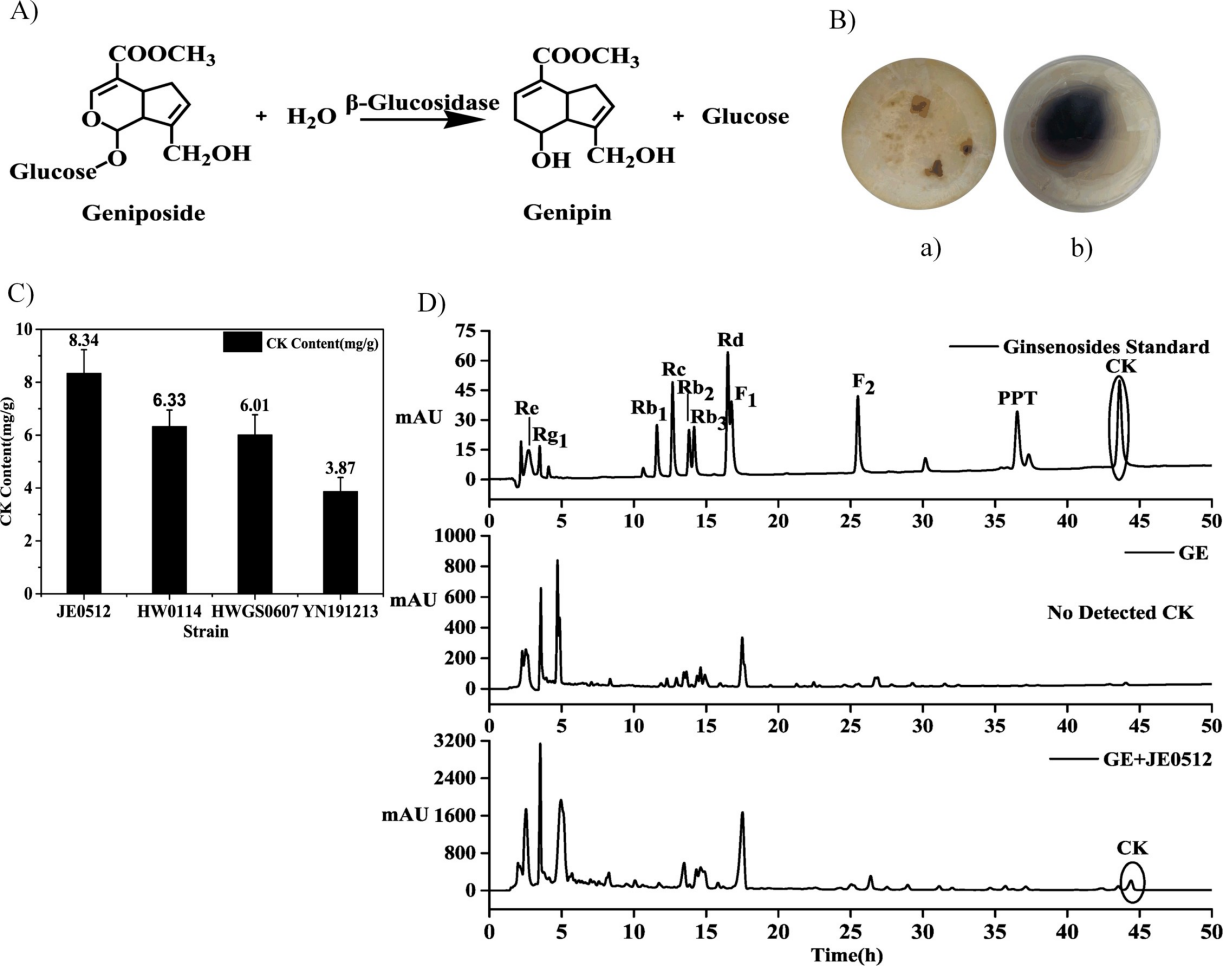
Screening of strains biotransformed GE into CK. A: Geniposide was hydrolyzed to genipin by β-glucosidase; B: Colonies on the plate without glucosidase activity (a), colonies with a blue circle had a higher β-glucosidase activity (b); C: Comparison the ability of strains to biotransform GE into CK. Values are means ± SD of three replications; D: HPLC profiles of ginsenosides in ginsenosides standard, GE, and GE fermented with JE0512.

Genipin, a hydrolysate of geniposide by β-glucosidase, produces a stable blue color upon reaction with amino acids [26] (Fig 2A). Based on this mechanism, β-glucosidase-producing strains were screened by plate coloration, colonies with a blue circle had a higher β-glucosidase activity (Fig 2Bb), while the negative colonies showed no color change (Fig 2Ba). We isolated 161 β-glucosidase producing microorganisms from the ginseng rhizosphere soils using the plate coloration method. Among them, four strains possessed the activity of transforming GE into CK as shown by HPLC analysis (Fig 2C and 2D), with JE0512 showing the highest activity in transforming GE into CK (8.34 mg/g). Fu et al. [23] isolated β-glycosidase-producing microorganisms from a ginseng field and found that *Sphingomonas asaccharolytica* could hydrolyze major ginsenosides to CK. There have been some previous reports on the use of GE as a substrate for the production of CK through fermentation. For example, Rae et al. [30] used *Cordyceps sinensis* to ferment red GE, and the CK content was 108.3 ± 13.5 μg/ml after 7 days. In another study, Kim et al. [31] fermented red GE with *Lactobacillus plantarum* M4, and the content of CK was 0.3 ± 0.05 mg/g in 5 days while Hsu et al. [20] obtained 1.11 ± 0.20 μmol/g of CK in 30 days by fermenting the American GE with *Ganoderma lucidum*. Compared with the above studies, JE0512 can obtain a higher yield in a short time. Therefore, JE0512 has great potential for the preparation of CK from GE.

### 3.2. UPLC-Q-TOF-MS analysis of a fermentation sample of JE0512

**Fig 3:**
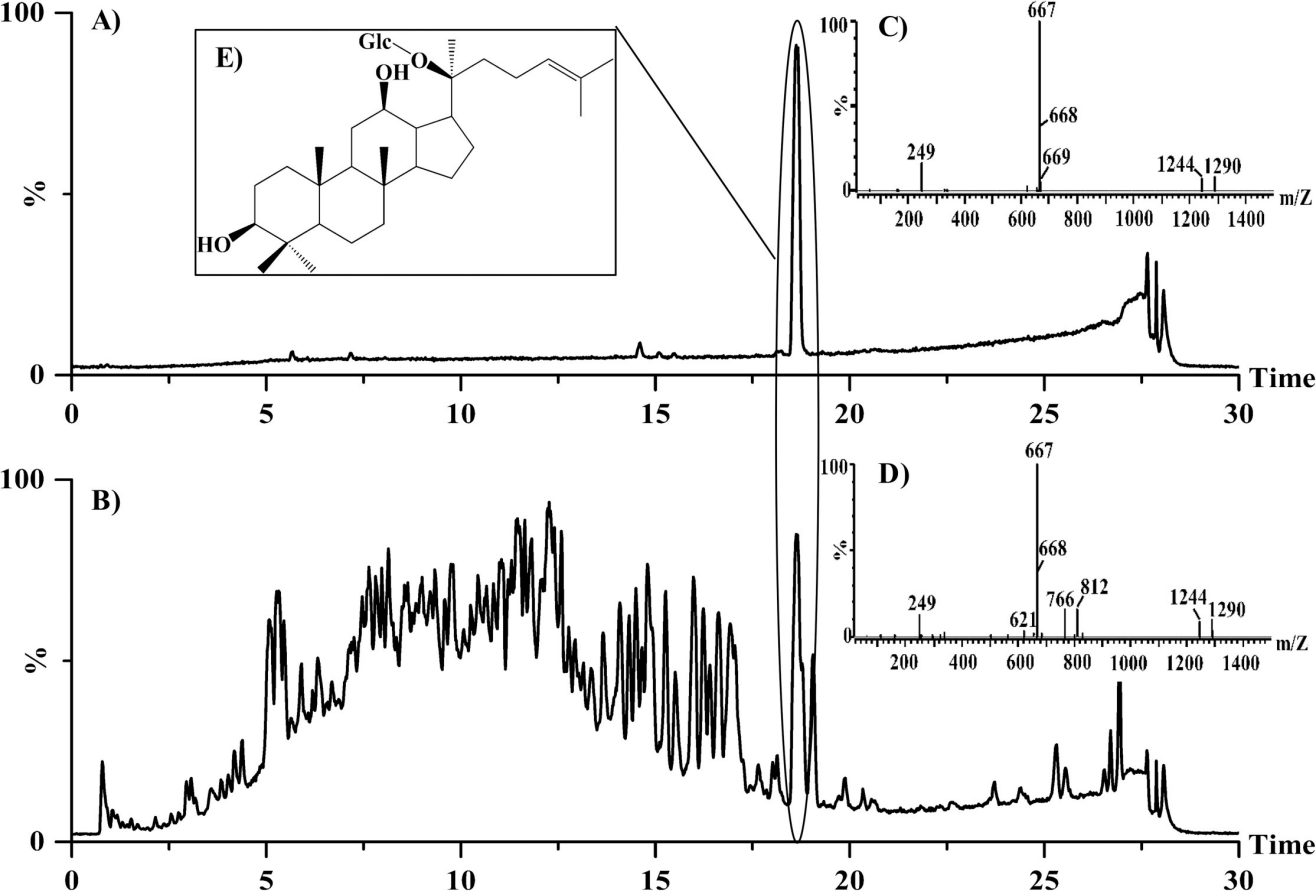
UPLC-Q-TOF-MS analysis of the fermented sample of JE0512. A: Total ion current chromatograms of CK standard; B: Total ion current chromatograms of JE0512 fermentation sample; C: Mass spectrum of CK standard; D: Mass spectrogram of JE0512 fermentation sample; E: The chemical structural formula of CK.

UPLC-Q-TOF-MS is one of the state-of-the-art techniques used for the rapid chemical profiling of medicinal herbs. Furthermore, the accurate mass values and low energy collision-induced dissociation (CID) of Q-TOF-MS have made UPLC-Q-TOF-MS a powerful tool for identifying and quantifying ginsenosides in ginseng [32]. CK is the major ginsenoside in the final fermentation product. Further confirmation and identification were carried out by comparing the mass spectra and retention times of samples collected from JE0512 (7 days) with the CK standard. The total ion current chromatograms of the CK standard (Fig 3A) showed a CK peak at approximately 18.61 min, while the total ion current chromatogram of the fermentation sample of JE0512 LSF had a clear peak at the same retention time (Fig 3B). This indicates that JE0512 can produce CK, which was supported by the HPLC detection of this product. The samples of the CK standard and JE0512 were analyzed by UPLC-Q-TOF-MS to determine the molecular weights. The CK standard samples showed a quasimolecular ion peak at m/z 667 [M–H + HCOOH] ^−^ (Fig 3C); however, an ion peak at m/z 621 [M − H] ^−^ (m/z 621) was not detected. The samples of JE0512 LSF showed quasi-molecular ion peaks at m/z 667 [M–H + HCOOH] ^−^ and m/z 621 [M − H] ^−^ (Fig 3D). Formic acid was added to the mobile phase, not only as a chromatographic modifier but also to produce [M–H + HCOOH] ^−^ adduction. For example, protopanaxadiol and protopanaxatriol-type ginsenosides could generate adduct ions [M–H + HCOOH] ^−^, which was helpful for the confirmation of deprotonated molecular ions [M − H] ^−^ [33]. The same retention time and fragment ions were used to confirm the existence of CK in the JE0512 fermentation samples.

### 3.3. Characterization of strain JE0512

**Fig 4:**
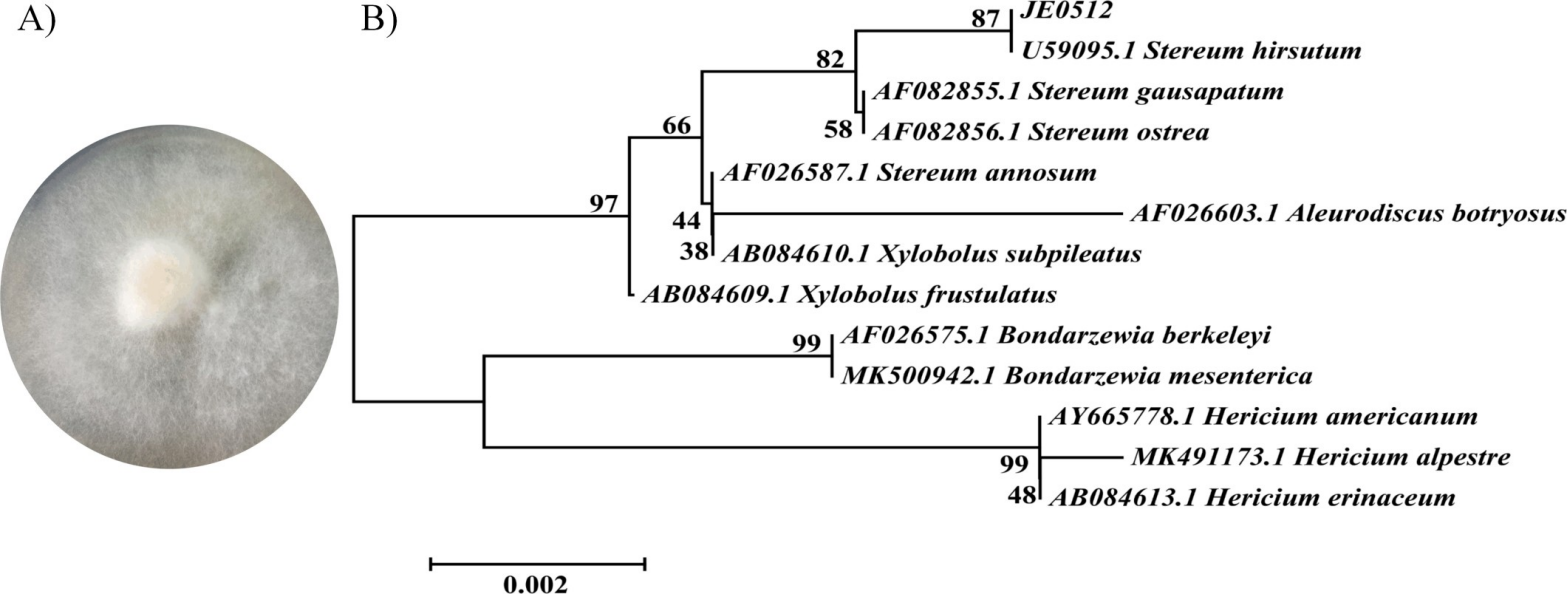
Identification of strain JE0512. A: Colony morphology of the isolate JE0512 grown at 25°C on PDA. B: Phylogenetic analysis of the related species of the strain JE0512 using the neighbor-joining approach. The scale bars represent 0.002 substitutions per site. The tree was constructed using a neighbor-joining method with bootstrap values of 1000 replications.

After culturing JE0512 at 25°C for 5–7 days on an agar plate, we characterized it as a filamentous, round, white fungus with a diameter of 4.0–7.0 cm (Fig 4A). A clamp connection was observed in the microstructure of the JE0512 mycelium, and the dense and reticular cross growth of hyphae could be seen in the scanning electron microscope (S1 Fig). For molecular characterization of strain JE0512, a 1,332-bp 18S rDNA fragment was amplified by PCR and sequenced. Sequence analysis indicated a 99.62% similarity with the sequence of *Stereum hirsutum* (accession no. U59095.1). A phylogenetic tree based on the 18S rRNA gene sequences of the isolate and phylogenetically related fungi (Fig 4B) confirmed that JE0512 is closely related to *Stereum hirsutum*. Thus, the research strain was identified as *Stereum hirsutum* JE0512. Fungi of the genus *Stereum* belong to the family *Stereaceae* (*Basidiomycetes*) [34]. Given that most of the *Stereum* species isolated from previous studies exhibited a series of interesting sesquiterpenoid skeletons, including sesquiterpene [35], benzoate ester [36], and norilludalane [37], this genus of fungi is one of the major sources of structurally diverse sesquiterpenes. To the best of our knowledge, there is no information on the biotransformation of GE to MGs by fungi belonging to the *Stereum* genus.

### 3.4. Optimization of main SSF process parameters for maximum CK content

According to the previous study [38,39], the fermentation substrate, inoculation volume, amount of GE, and fermentation period are among the main parameters influencing CK content and β-glucosidase activity in the SSF process. The optimum values of these parameters to maximize CK content and β-glucosidase activity when fermented by *Stereum hirsutum* were determined using a single-factor methodology. The results are presented in this section.

The optimal SSF process parameters for CK content were 20 g corn bran substrate, 4 g GE, and 20% inoculation volume.

#### 3.4.1. Optimization of agro-industrial substrates

**Fig 5:**
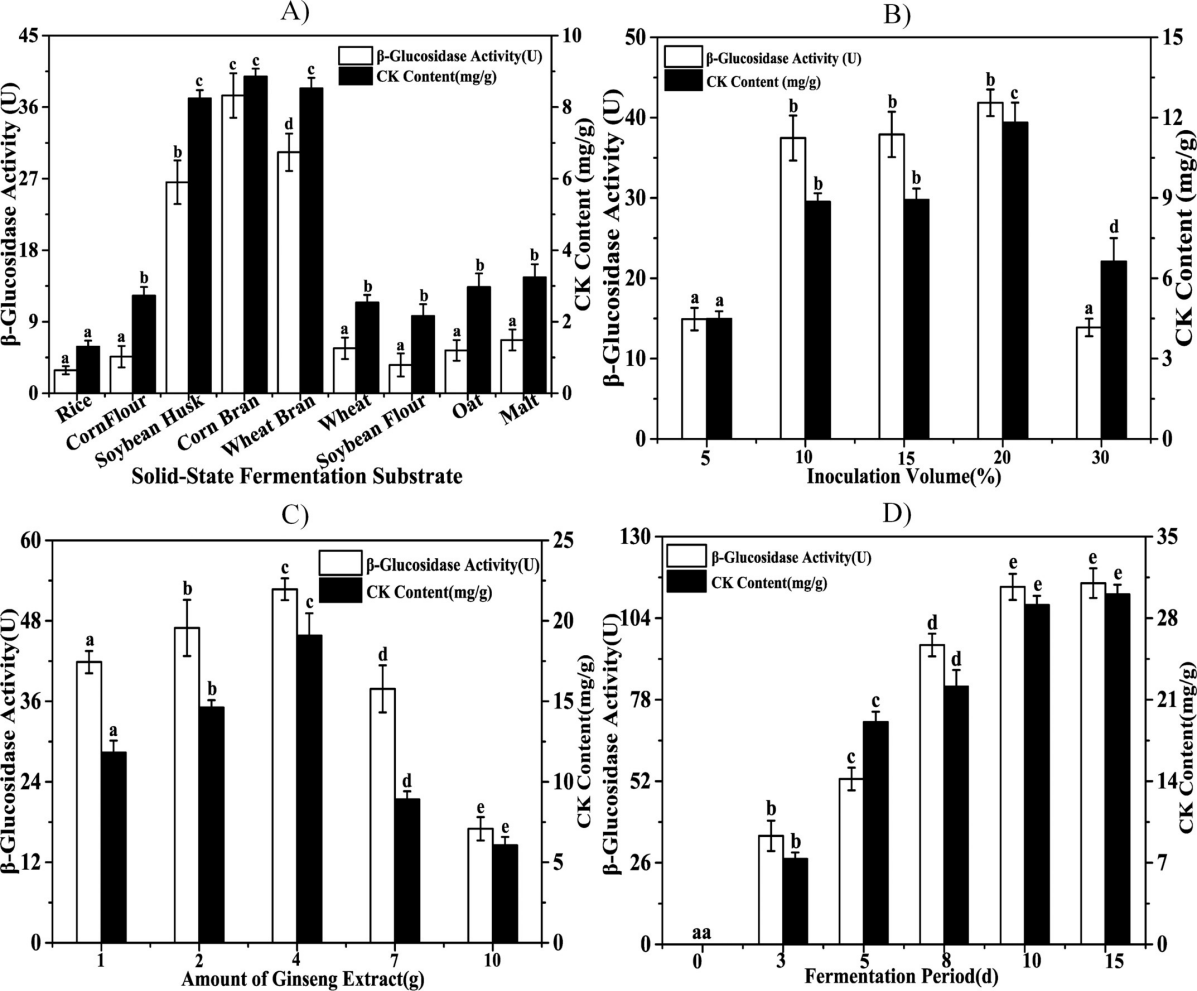
Optimization of main SSF process parameters for maximum CK content and β-glucosidase activity. A: Effect of the solid-state fermentation substrate (20 g); B: Effect of inoculation volume (%); C: Effect of the amount of ginseng extracts (g); D: Effect of fermentation period (d). Values are shown as means ± SD of three replications. A one-way ANOVA was used to assess the statistical significance of the differences in expression levels. Different letters (a-e) indicate significant differences between each other (P < 0.05).

The production yields of secondary metabolites can be improved with SSF, and the solid substrate is a key factor for the efficient and economical production of target bioproducts [40]. Thus, the selection of an appropriate solid substrate is of great importance for industrial production. In this study, different agro-industrial substrates, including rice, cornflour, soybean husk, corn bran, wheat bran, wheat, soybean flour, oat, and malt, were examined for their ability to support SSF. Because of starch gelatinization, starchy substrates usually exhibit high viscosity after high-temperature sterilization [41]. Taking the high O_2_ consumption in CK production in SSF into consideration, porous substrates could improve the conditions of aeration and oxygen transfer. As shown in Fig 5A, CK content in SSF with soybean husk (8.24 mg/g), corn bran (8.86 mg/g), and wheat bran (8.52 mg/g) as the substrate were significantly higher than that with other substrates. These are cost-effective and easily available renewable resources. However, their potential has not been fully realized. This study provides a new idea for their utilisation. Rice, soybean flour, and other substrates have a high starch content, which increases the viscosity and volume of substrates after sterilisation at high temperature and high pressure, thus decreasing the gap between particles and the dissolved oxygen coefficient. It is not conducive to the spread and growth of mycelia; however, this problem can be overcome by the addition of corn bran.

Filamentous fungi, yeasts, and bacteria are widely used to produce enzymes by SSF. SSF is an attractive method for filamentous fungus cultivation because the solid substrates mimic the natural habitat of fungi, resulting in improved growth and secretion of a wide range of enzymes [42]. The conversion of enzymatic ginsenoside-to-CK is greatly affected by enzyme activities, and β-glucosidase activity is considered the key factor in the selection of strains to be used for biotransformation of ginsenosides to CK [43]. In this study, β-glucosidase was selected to evaluate the enzyme activity during SSF among other enzyme activities in *Stereum hirsutum* JE0512. The SSF β-glucosidase activities of soybean husk (26.55 U), corn bran (37.46 U), and wheat bran (30.30 U) were significantly higher than those of other substrates (Fig 5A) and were consistently correlated with CK content. Corn bran was the best substrate for both CK content and β-glucosidase activity and provided the best support among the nine tested agro-industrial substrates. From these results, corn bran was selected as the optimal substrate for SSF in subsequent experiments, it is very high in fiber, and it can be used in a wide range of processes. Many commercial food producers use this substance as a filler in their products, and to reduce the caloric value of snack foods. The edibility of the substrate makes it possible for us to study the use of this technology for the production of snack foods or health products containing CK in the later stage.

#### 3.4.2. Optimization of inoculum concentration

Fig 5A shows the proportional relationship between CK content and β-glucosidase activity. We also found a proportional relationship between fungal biomass and β-glucosidase activity in our initial experiments. Based on these results, we determined the effect of inoculum concentration on CK content and β-glucosidase activity. *Stereum hirsutum* JE0512 was cultured in 20 g corn bran substrate and 1g GE at 25°C for 5 d. The effects of different inoculation volumes on CK content and β-glucosidase activity are shown in Fig 5B. Our results confirmed that CK content and β-glucosidase activity increased slightly following the increase in inoculation volume. However, CK content and β-glucosidase activity were reduced at a 30% inoculation volume. These results also indicate that an inoculation volume of 20% was optimal for enzyme production and enhanced ginsenoside production. The maximized CK content and β-glucosidase activity was 11.82 mg/g and 41.85 U, respectively. Logically, the enzyme activity should increase with an increase in inoculation volume and biomass. The maximum enzyme activity was not reached at an inoculation volume of 30%, as the excess fungal biomass consumed nutrients too quickly.

#### 3.4.3. Optimization of GE amount

To investigate the effect of the amount of GE on enzyme activity and CK content, 20% inoculation volume of *Stereum hirsutum* JE0512 was cultured in 20 g corn bran substrate with different amounts of GE (1 g, 2 g, 4 g, 7 g, and 10 g), where the optimal amount of GE for CK production and β-glucosidase activity was found to be 4 g (Fig 5C). The highest CK content was 19.09 mg/g, and the highest β-glucosidase activity was 52.71 U. In our initial experiments, we found that GE has a high viscosity. Therefore, we hypothesize that adding an appropriate amount of extract would help the production of enzymes while adding an excessive amount of GE would increase the viscosity of the fermentation substrate and delay the growth of mycelium. The optimal amount of GE we mention here provides a reference for the development of *Stereum hirsutum* JE0512 for the industrial production of CK.

#### 3.4.4. Optimization of the fermentation period

The effect of the fermentation period on the biotransformation process was investigated under optimized fermentation conditions (20 g corn bran substrate, 4 g GE, and 20% inoculation volume). A bar chart of the samples at different days of fermentation is shown in Fig 5D. Our results indicated that CK content and β-glucosidase activity increased significantly with the prolongation of the fermentation period. Using unfermented GE and medium as the control, the CK content and β-glucosidase activity in the samples of the control group could not be detected. After 10 days of fermentation, we observed that the composition of ginsenosides was significantly altered; while the content of CK increased from 8.86 mg/g to 29.13 mg/g (4-fold increase), the activity of β-glucosidase increased from 37.46 U to 113.96 U (3-fold increase). There was no significant change in CK content and β-glucosidase activity at 15 days compared to the 10 days. We chose to ferment for 10 days for a greener economy.

### 3.5. Analysis of the effects of combining JE0512 with cellulase

The optimal operational conditions vary over a wide range depending on the process, and different products can be obtained from the same substrate under different operational conditions and/or by inoculating different strains of microorganisms [42]. The industrially important MGs can be efficiently produced from major ginsenosides by the combined use of different-type ginsenosidases [44]. Therefore, we combined *Stereum hirsutum* JE0512 with cellulases from different sources (*Trichoderma viride* and *Aspergillus niger*) to analyze the changes in ginsenoside content produced during the fermentation process. In this study, 4 g of GE and 2.5% (w/w) cellulase were added to 20 g corn bran substrate with a 20% inoculation volume, and the changes in ginsenoside content affected by different cellulases were investigated. The changes in ginsenoside content in GE, GE + JE0512, GE + JE0512 + cellulase (*T*. *viride*), and GE + JE0512 + cellulase (*A*. *niger*) were analysed (Table 1). 11 ginsenosides (Rb_1_, Rc, Rb_2_, Rb_3_, Rd, F_2_, Re, Rg_1_, F_1_, CK, and PPT) were analysed by HPLC.

**Table 1:** The changes in ginsenosides content under different fermentation methods.

Ginsenoside	Ginsenoside content (mg/g)			
	GE (Control)	GE + JE0512	GE + JE0512 + Cellulase (*T*. *viride*)	GE+ JE0512 + Cellulase (*A*. *niger*)
**Rb**_**1**_	16.28 ± 0.03 ^a^	n. d. ^b^	n. d. ^b^	n. d. ^b^
**Rc**	28.00 ± 0.03 ^a^	4.98 ± 2.31 ^b^	3.21 ± 2.01 ^b^	n. d. ^c^
**Rb**_**2**_	65.16 ± 0.01 ^a^	18.98 ± 2.79 ^b^	17.70 ± 2.37 ^b^	47.31 ± 2.92 ^c^
**Rb**_**3**_	38.74 ± 0.04 ^a^	28.58 ± 3.45 ^b^	29.12 ± 2.25 ^b^	n. d. ^c^
**Re**	n. d. ^a^	n. d. ^a^	n. d. ^a^	n. d. ^a^
**Rg**_**1**_	178.27 ± 0.01 ^a^	n. d. ^b^	n. d. ^b^	n. d. ^b^
**F**_**1**_	n. d. ^a^	n. d. ^a^	n. d. ^a^	n. d. ^a^
**Rd**	270.24 ± 0.02 ^a^	160.76 ± 3.33 ^b^	159.88 ± 2.96 ^b^	79.79 ± 3.27 ^c^
**F**_**2**_	1.66 ± 0.02 ^a^	11.17 ± 2.64 ^b^	8.26 ± 2.45 ^c^	23.39 ± 3.52 ^d^
**CK**	n. d. ^Ca^	29.13 ± 3.78 ^b^	30.03 ± 2.36 ^b^	54.48 ± 3.11 ^c^
**PPT**	n. d. ^a^	13.74 ± 3.19 ^b^	16.04 ± 2.17 ^c^	52.92 ± 2.09 ^d^
**Rb**_**1**_ **+ Rc + Rb**_**2**_ **+ Rb**_**3**_ **+ Re + Rg**_**1**_ **+ F**_**1**_ **+ Rd** ^**A**^	596.69 ± 0.14 ^a^	213.30 ± 11.88 ^b^	209.91 ± 9.59 ^b^	127.1 ± 6.19 ^c^
**F**_**2**_ **+ CK + PPT** ^**B**^	1.66 ± 0.02 ^a^	54.04 ± 9.61 ^b^	54.33 ± 6.98 ^b^	130.79 ± 8.72 ^c^

Values are shown as means ± SD of three replications. A one-way ANOVA was used to assess the statistical significance of the differences in expression levels. Means with different small letters (a-e) in the same line are significantly different (p < 0.05).

A: Total content of Rb_1_ + Rc + Rb_2_ + Rb_3_ + Re + Rg_1_ + F_1_ + Rd.

B: Total content of F_2_ + CK + PPT.

C: No detected.

Our results indicated that the amounts of ginsenosides Rb_1_, Rc, Rb_2_, Rb_3_, Re, Rg_1_, F_1,_ and Rd (596.69 ± 0.14 mg/g) in uninoculated GE (control) were higher than the amounts of MGs F_2_, CK, and PPT (1.66 ± 0.02 mg/g), with Rd (270.24 ± 0.02 mg/g) being the highest. The content of Rb_1_, Rc, Rb_2_, Rb_3_, Re, Rg_1_, F_1_ and Rd in GE was 16.28, 28.00, 65.16, 38.74, 0.00, 178.27, 0.00, 270.24 ± 0.02 mg/g respectively. After GE was fermented, none of the samples from three fermentation methods [(GE + JE0512, GE + JE0512 + cellulase (*T*. *viride*), and GE + JE0512 + cellulase (*A*. *niger*)] had detectable levels of ginsenosides Rb_1_, Re, and Rg_1_, which were biotransformed into more bioactive MGs [45] (F_2_, CK, and PPT) (S2 Fig). It is well known that microorganisms and/or their enzymes convert ginsenosides to CK through the hydrolysis pathways of Rb_1_→Rd→F_2_→CK [23], Rc→Gyp XVII→Gyp LXXV→CK [46], Rb_2_→CO→CY→CK [23], Rb_3_→CMx_1_→CMx→CK [23], and Rc→CMc_1_→CMc→CK [47], and PPT through the hydrolysis pathways of Re→Rg_1_→F_1_→PPT, Re→Rg_2_→Rh_1_→PPT, and Rf→Rh_1_→PPT [48] (S3 Fig). SSF offers many advantages over LSF, such as a low risk of contamination, high yield, and high enzyme activity, which make this process more attractive [42]. The experimental data we obtained verify this theory. After 10 days of fermentation, the composition of ginsenosides was significantly altered, the content of ginsenosides (Rb_1_, Rc, Rb_2_, Rb_3_, Re, Rg_1_, F_1_ and Rd) decreased from 596.69 mg/g (GE) to 213.30 mg/g (GE + JE0512), while the content of MGs (F_2_, CK and PPT) increased from 1.66 mg/g (GE) to 54.04 mg/g (GE + JE0512). Among them, ginsenoside Rd showed the highest decrease, 109.48 mg/g, and CK showed the highest increase, of 29.13 mg/g. CK is the major ginsenoside in the final fermentation product, and the contribution of CK to the total amount of MGs (F_2_, CK, and PPT) was 53.90%. Therefore, we suggest that the increase in ginsenoside CK level was mainly caused by the conversion of Rd during fermentation. Additionally, the contents of the other four ginsenosides (Rb_1_, Rc, Rb_2_, and Rb_3_) also decreased during fermentation. Taken together, the increase in CK level may be mainly attributed to the conversion of Rd by the removal of two glucose units at the C-3 position. Furthermore, ginsenoside Rb_1_ can be transformed into CK, by the removal of two glucose units at the C-3 position and one glucose unit at the C-20 position, while Rc can be transformed into CK, by removing two glucose units at the C-3 position and one arabinose unit at the C-20 position, Rb_2_ can be transformed into CK, by removing two glucose units at the C-3 position and one arabinose unit at the C-20 position, Rb_3_ can be transformed into CK, by removing two glucose units at the C-3 position and one xylose unit at the C-20 position, and Re can be transformed into PPT, by removing one glucose unit and one rhamnose unit at the C-6 position and one glucose unit at the C-20 position. The reactions can be summarised as follows: Rb_1_→Rd→F_2_→CK, Rc→Gyp XVII→Gyp LXXV→CK, Rb_2_→CO→CY→CK, Rb_3_→CMx_1_→CMx→CK, and Re→Rg_1_→F_1_→PPT (S3 Fig).

In samples with GE + JE0512 + cellulase (*T*. *viride*), the MGs (F_2_, CK, and PPT) content was not significantly different from those of JE0512. These results indicate that cellulase (*T*. *viride*) had almost no effect on the total ginsenoside content. It has been reported that enzymes belong to glycoside hydrolase (GH) family 3 can hydrolyze the inner glucose at C-20, C-3, and C-6 of ginsenosides, and enzymes belong to GH family 1 can hydrolyze the outer glucose at C-20 and C-3 in PPD-type ginsenosides [49]. In addition, arabinosidases, rhamnosidases, xylosidases, and β-glycosidase belong to the GH family 51, 78, 39, 2, and 42 can act on the deglycosylation reaction of ginsenosides. Since enzymes are substrate specific, and we speculate that cellulase from *T*. *viride* cannot act on the deglycosylation reaction of ginsenosides because the GH family to which they belong may not have the ability to hydrolyze the glycosyl group of ginsenosides. On the other hand, we added cellulase (*A*. *niger*) during the fermentation of *Stereum hirsutum* JE0512. The CK content of GE + JE0512 + Cellulase (*A*. *niger*) was higher than that found by the other fermentation methods. These results indicate that the content of MGs (F_2_, CK, and PPT) in the GE + JE0512 + cellulase (*A*. *niger*) fermentation method (130.79 mg/g) was higher than that of other fermentation methods (Table 1). In addition, the CK content of GE + JE0512 + cellulase (*A*. *niger*) (54.48 mg/g) was approximately 2-fold that of JE0512 (29.13 mg/g), while the content of Rb_1_, Rc, Rb_3_, and Rd significantly decreased. Among them, ginsenoside Rd showed the greatest decrease, and Rb_1_, Rc, and Rb_3_ were all converted to MGs (F_2_, CK, and PPT). Additionally, the PPT content (52.92 mg/g) was greater than that in the other fermentation methods. The results indicated that SSF combined with cellulase (*A*. *niger*) and *S*. *hirsutum* enhances the conversion of ginsenosides into MGs. The different types of saponin hydrolases produce different deglycosylated ginsenosides from glycosylated ginsenosides by specifically hydrolyzing the glycosyl group of the ginsenosides [49]. The production of deglycosylated ginsenosides can be efficiently achieved by combining different types of ginsenoside hydrolases. This study combined high-glucosidase-producing strain *S*. *hirsutum* with cellulase (*A*. *niger*) in SSF to provide a reference for the industrial production of MGs. HPLC chromatograms of GE and samples from three different fermentation methods are shown in S2 Fig. The results indicated that the CK content of GE + JE0512 + cellulase (*A*. *niger*) was higher than that of other fermented methods.

Compared with major ginsenosides, MGs are more easily digested and absorbed by the body due to their lower molecular weight and membrane permeability, which will increase the nutritional value of healthy food if they contain multiple MGs. Furthermore, the fermented mycelia of *S*. *hirsutum* have been reported to abundantly produce active secondary metabolites in recent years, which have antibacterial activities [50] and the ability to inhibit α-glucosidase [51]. However, there are few studies on the active metabolites of *S*. *hirsutum* compared to other edible and medicinal mushrooms. These findings in this study are hoped to provide a reference for other researches studying *S*. *hirsutum*. Thus, fermented products contain a variety of MGs and multiple active metabolites of *S*. *hirsutum*, which have the potential to be used as dietary foods.

## 4. Conclusions

In conclusion, this study found that *Stereum hirsutum* could transform GE into MGs (F_2_, CK, and PPT). After combining *Stereum hirsutum* JE0512 with cellulase (*Aspergillus niger*) in SSF using agro-industrial waste as substrate, CK content increased from 8.34 mg/g to 54.48mg/g (79-fold increase) and the MGs (F_2_, CK, PPT) content increased from 1.66 mg/g to 130.79 mg/g (79-fold increase) in the final product. The findings of this study provide a reference for the circular bioeconomical production of bioactive products containing a variety of MGs and offer a means for the development of new technologies for fermenting *Stereum hirsutum* to prepare a variety of high-value healthy products.

## Supporting information

S1 Fig Morphological features of JE0512. A: Colony morphology of the isolate JE0512 grown at 25°C on PDA; B: The figure of clamp connection by optical microscope (10×); C: Scanning electron micrograph of mycelia (600×).
(TIF)

S2 Fig The liquid chromatogram of different fermentation methods. (TIF)

S3 Fig The biotransformation pathway of ginsenosides. A, the biotransformation pathway of prototopanaxadiol types of ginsenosides to CK [5–7]; B, the biotransformation pathway of protopanaxatriol types of ginsenosides to PPT [8].
(TIF)

S1 Table Related reports on the preparation of ginsenoside CK from GE. (DOCX)
