# Supplementary figures and images for: Production of minor ginsenosides by combining Stereum hirsutum and cellulase
Source: PLoS One. 2021 Aug 6;16(8):e0255899. doi: 10.1371/journal.pone.0255899 (PMC8345839; doi:10.1371/journal.pone.0255899)

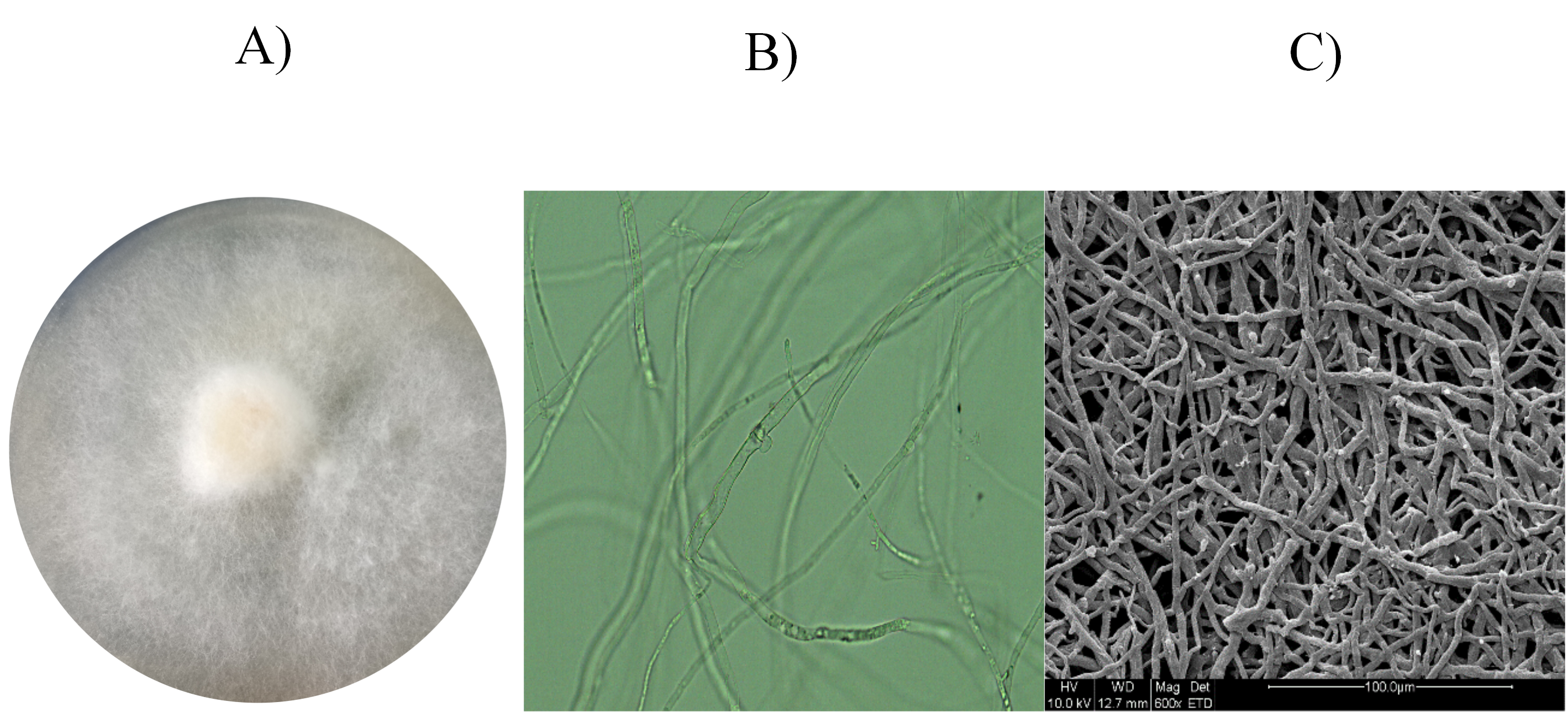

Supplement: S1 Fig — A: Colony morphology of the isolate JE0512 grown at 25°C on PDA; B: The figure of clamp connection by optical microscope (10×); C: Scanning electron micrograph of mycelia (600×). (TIF) [file pone.0255899.s001.tif]

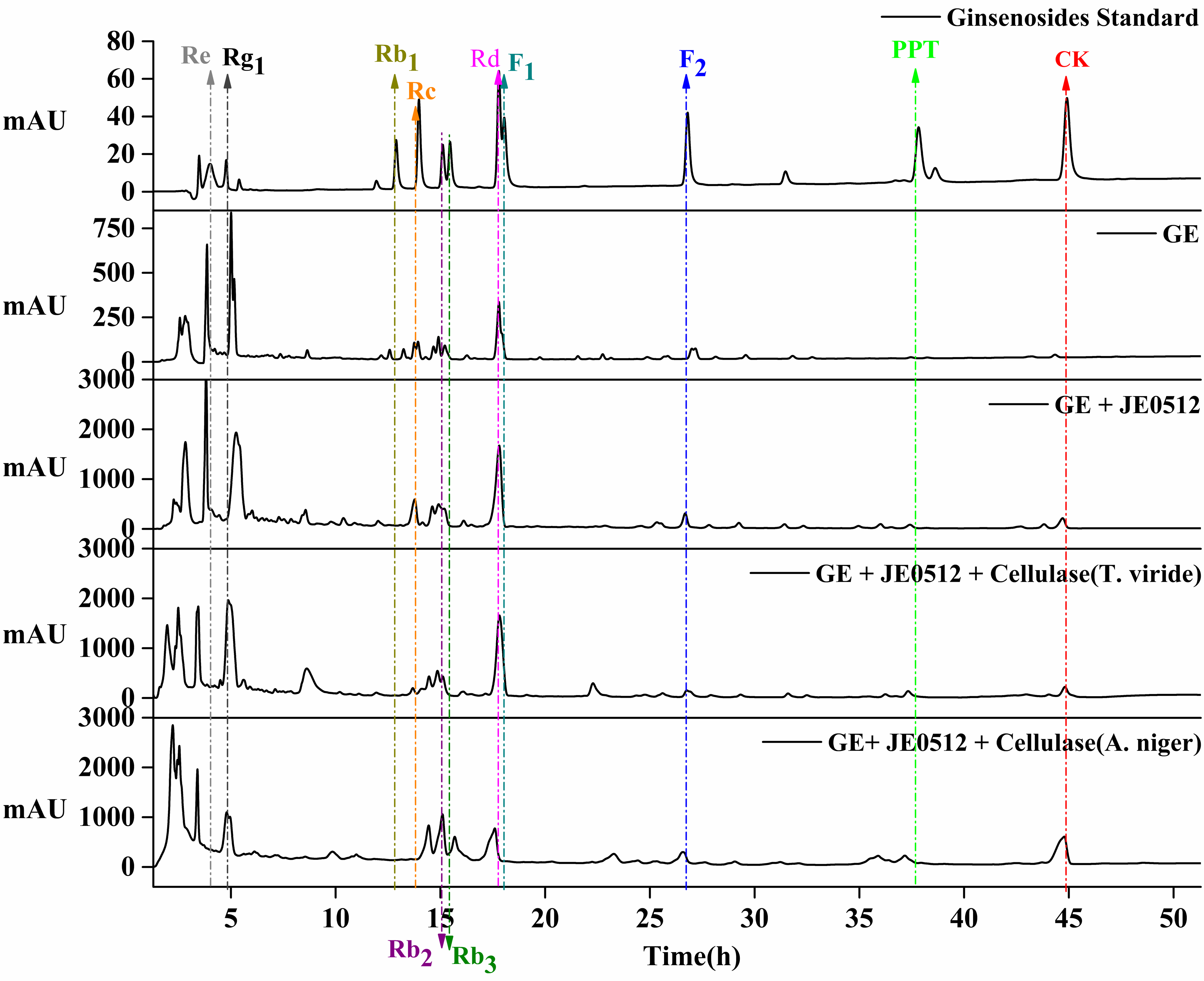

Supplement: S2 Fig — (TIF) [file pone.0255899.s002.tif]
