# Supplementary material for: Production of minor ginsenosides by combining Stereum hirsutum and cellulase
Source: PLoS One. 2021 Aug 6;16(8):e0255899. doi: 10.1371/journal.pone.0255899 (PMC8345839; doi:10.1371/journal.pone.0255899)
Supplement: S1 Table — (DOCX) [file pone.0255899.s004.docx]

**Table S1.** Related reports on the preparation of ginsenoside CK from ginseng extract.

| Strain | Substrate | CK content in 250 mL shake flask | Fermentation time |
| --- | --- | --- | --- |
| *Cordyceps sinensis*  [1] | Red ginseng extract | 108.3 ± 13.5 μg / mL | 7 days |
| *Lactobacillus brevis*  [2] | Ginseng extract | About 0.32 mg / g | 5 days |
| *Ganoderma lucidum*  [3] | The American ginseng extraction residue | 1.11 ± 0.20 μmole / g | 30days |
| *Lactobacillus plantarum* M4  [4] | Red ginseng extract | 0.3 ± 0.05 mg / g | 5 days |

1. Rae SH, Lee H-S, Kim M-R, Kim SY, Kim J-M, Suh HJ. Changes of Ginsenoside Content by Mushroom Mycelial Fermentation in Red Ginseng Extract. Journal of Ginseng Research. 2011;35(2):235-42. doi: <https://doi.org/10.5142/jgr.2011.35.2.235>.

2. Yoo J-M, Lee JY, Lee YG, Baek S, Kim MR. Enhanced production of compound K in fermented ginseng extracts by Lactobacillus brevis. Food Sci Biotechnol. 2019;28(3):823-9. doi: <https://doi.org/10.1007/s10068-018-0504-0>

3. Hsu BY, Lu TJ, Chen CH, Wang SJ, Hwang LS. Biotransformation of ginsenoside Rd in the ginseng extraction residue by fermentation with lingzhi (Ganoderma lucidum). Food Chem. 2013;141(4):4186-93. doi: <https://doi.org/10.1016/j.foodchem.2013.06.134>.

4. Kim B-G, Choi S-Y, Kim M-R, Suh HJ, Park HJ. Changes of ginsenosides in Korean red ginseng (Panax ginseng) fermented by Lactobacillus plantarum M1. Process Biochemistry. 2010;45(8):1319-24. doi: <https://doi.org/10.1016/j.procbio.2010.04.026>.
